# What Not to Expect from Koch

**Published:** 1890-11-08

**Authors:** 


### The Hospital

A Weekly Journal of

**Science, Medicine, Nursing, and Philanthropy.**

*For Hospitals, Asylums, Infirmaries, Dispensaries, Medical Practitioners, Students, Nurses, and the Charitable Public.*

Vol. IX.—No. 215.] [November 8, 1890.

### What Not to Expect from Koch.

Not only the medical profession, but every newspaper correspondent in the civilized world is waiting for the announcement of Dr. Koch's successful cure of the universally dreaded consumption. At Berlin the chief subject of conversation in medical circles is Koch's experiments. There is not much food for discussion, because the great professor keeps all his operations as strictly secret as if he were planning the overthrow of a dynasty. It is believed, however, that the new remedy for consumption will not be a drug which can be purchased at the chemists' shop. The Alpha and the Omega of the method will, it is inferred, consist in the destruction of the tubercle bacillus, and the consequent arrest of the phthisical process in the lungs. This shows the experienced physician exactly what to expect from Professor Koch, and what not to expect.

Practical persons will do well to bear in mind the very considerable variety of conditions included under the term phthisis. Which of those conditions does Dr. Koch intend to deal with and to cure; or does he expect to cure them all? To the public a consumptive person is one already more than half dead from fever, bloodlessness, weakness, and disintegrated lungs. Are all such persons, when properly placed under Dr. Koch's treatment, to begin to recover immediately, and to finally regain their primitive health and strength? It is much to be feared that the world at large is looking for a result of this kind: it is equally certain that such an expectation is doomed to disappointment: and it is not improbable that, however successful Koch's treatment may be in destroying the tubercle bacillus, its failure to restore all consumptives to health will give occasion to sceptics to repeat their derisive criticisms about medical promises and their non-fulfilment in definite and positive cures.

It is on occasions like the present that the scientific practitioner so bitterly deplores the complete ignorance of even the most educated portion of the public of the nature of disease and of the rational possibilities of cure. Dr. Koch may show to the world a discovery of the most far-reaching consequences, and of the first order of importance; and it may happen that, because it does not produce immediately those particular results which an uninstructed public are looking for, he may receive little but derision for his reward. What is phthisis, and what is the tubercle bacillus? The general reader imagines them to be practically one and the same thing. They are no more one and the same thing than an acorn and an oak are one and the same thing. It is easy to destroy an acorn; but unless a man have fire at his disposal, how is he going to set about destroying an oak? So also a child may kill a newly-born lion's whelp without the least difficulty; but what man, destitute of fire-arms, will vanquish the full-grown lion? Or, still more, how will he restore to life and activity the cattle, the deer, and possibly the human beings which the lion may have devoured in the course of a long lifetime? These illustrations do not at all over-state the difference between the condition found on the first entrance of the tubercle bacillus into the human lungs, and the desolating ravages produced by it in the course of a few weeks' or months' activity. The most ignorant may see that whilst it may be a comparatively easy task to destroy tubercle bacilli when they are few, and to repair the damage done by them when it is still slight, it may be extremely difficult, and even impossible, to extirpate them when they are present in millions, and to rebuild the once fair house whose walls and roof they have laid level with the ground.

Hitherto we have written as if the tubercle bacillus were at least the chief, even if not the only cause of phthisis. But many of the most competent British practitioners are not at all convinced that the tubercle bacillus is the only or the chief cause of phthisis. Some of them go so far as to doubt whether it is a cause at all. That rod-shaped bacteria are almost constantly associated with phthisis is unquestionable; that they are associated with it as the chief or only cause has yet to be proved. Both medical and lay readers will do well to consider the following quotation from the new edition (1890) of "Quain's Dictionary of Medicine": "The conditions which give rise to phthisis are varied and diverse; but by tracing out their mode of action on the individual, we arrive at a twofold arrangement into general and local causes. In the general the constitution of the individual and the functions of nutrition and assimilation appear to be first involved. In the local the lungs are the primary seat of disease. The general may be called constitutional, and the local inflammatory. General causes are those which affect the whole system, such, for example, as family predisposition; fevers and exanthemata; syphilis; insufficient food; alcohol; bad ventilation; climatic influences; dampness of soil; infection; and so on. Amongst local causes are to be enumerated inflammatory affections of the lungs and pleura; trades and occupations giving rise to a gritty atmosphere; and injuries to the chest."

Now those who desire to think like men on this subject, and not like feeble enthusiasts whose faith is always that of the latest and boldest speaker, will do well to note one particular fact in the foregoing brief description: it is this, that whilst thirteen causes of phthisis are here given, only one of them is of the kind that implies a definite bacillary origin of the disease. That one, of course, is infection. On the point of infection we will again quote Quain's Dictionary, "Experiments have been carried on by numerous observers to ascertain whether tubercle is or is not inoculable; and the results of these experiments prove that in guinea pigs and rabbits tubercle can be produced artificially by the insertion underneath the skin, not only of tubercle, but of various other materials, such as pus, putrid muscle, and diseased liver taken from non-tuberculous subjects. There was nothing specific in the results of the inoculations, for the materials most efficient in producing artificial tubercule were those taken from low pneumonia, pyæmic abscess, etc., while human tubercle, phthisical sputa, foul pus, and putrid muscle were less successful. . . It was found by Dr. Burdon Sanderson that tuberculosis might be induced in the guinea-pig by the insertion of a cotton thread under the skin." We have not space here even to indicate the varied significance of facts like these; but one thing is plain, that they seem to show in the most convincing way that tuberculosis is not a simple disease, having always one and the same cause, and that cause a definite and decisively ascertained living organism. Scarlatina, we believe, always springs from pre-existing scarlatina, and does not arise in any other way. The same may, of course, be said of several other diseases whose names will at once occur to the medical mind. But if the present state of medical knowledge in this country be at all representative of the facts, the same cannot be said of tuberculosis.

There are those who think that whilst medical knowledge on this subject is confused and doubtful in this country it is clear and luminous in Germany. That we hold to be the most absolute nonsense. The Germans we believe to be the rarest fellows in the world for scenting a cunning fox and running him down to the death. But it has very often happened in every department of their scientific and literary activity that their fox has been a weasel, and sometimes nothing better than a fresh badger's skin drawn by mischievous hands over the dewy ground. In estimating the value of German scientific discoveries this characteristic of the national intellect is not to be forgotten. Demonstrable medical science is not more advanced in Germany, France, or America than in this country. On the contrary, we have in Great Britain an army of physicians and surgeons who are in the very forefront of practical knowledge and skill. Looking at the probabilities of success which lie before Professor Koch from the British standpoint, we feel that there is an urgent necessity for uttering both to the profession and to the public a note of warning. In common with all classes of the people in every civilized country we await with lively hope the announcement which any day may bring forth; but whilst we hope, and hope fervently, reason suggests that, if we would avoid the bitterness of disappointment, we must not expect too much.

